# The effectiveness of water treatment processes against schistosome cercariae: A systematic review

**DOI:** 10.1371/journal.pntd.0006364

**Published:** 2018-04-02

**Authors:** Laura Braun, Jack E. T. Grimes, Michael R. Templeton

**Affiliations:** Department of Civil and Environmental Engineering, South Kensington Campus, Imperial College London, London, United Kingdom; University of Florida, UNITED STATES

## Abstract

**Background:**

Schistosomiasis is one of the most disabling neglected tropical diseases, ranking second in terms of years lived with disability. While treatment with the drug praziquantel can have immediate beneficial effects, reinfection can occur rapidly if people are in contact with cercaria-infested water. Water treatment for schistosomiasis control seeks to eliminate viable cercariae from water, thereby providing safe alternative water supplies for recreational and domestic activities including laundry and bathing. This provision may reduce contact with infested water, which is crucial for reducing reinfection following chemotherapy and cutting schistosome transmission.

**Methodology:**

A qualitative systematic review was carried out to summarize the existing knowledge on the effectiveness of water treatment in removing or inactivating human schistosome cercariae. Four online databases were searched. Studies were screened and categorized into five water treatment processes: storage, heating, chlorination, filtration, and ultraviolet (UV) disinfection.

**Conclusions:**

All five water treatment methods can remove or inactivate cercariae in water, and hence produce cercaria-free water. However, reliable design guidelines for treating water do not exist as there are insufficient data. Overall, the review found that cercariae are inactivated when storing water for 10–72 hours (depending on temperature), or with chlorination values of 3–30 mg-min/l. UV fluences between 3–60 mJ/cm^2^ may significantly damage or kill cercariae, and sand filters with 0.18–0.35 mm grain size have been shown to remove cercariae. This systematic review identified 67 studies about water treatment and schistosomiasis published in the past 106 years. It highlights the many factors that influence the results of water treatment experiments, which include different water quality conditions and methods for measuring key parameters. Variation in these factors limit comparability, and therefore currently available information is insufficient for providing complete water treatment design recommendations.

## Author summary

Schistosomiasis control currently focuses on preventive chemotherapy (PC) with praziquantel, which is effective, safe, and inexpensive. However, this treatment does not prevent subsequent reinfection. As schistosomiasis control targets become more ambitious and move towards elimination, interest is increasing in the potentially complementary roles of water, sanitation, and hygiene (WASH) interventions which may disrupt transmission of the parasite, thereby slowing reinfection following treatment. Water treatment for schistosomiasis control seeks to eliminate viable schistosome cercariae from water. We carried out a systematic review to summarize the existing knowledge on the effectiveness of water treatment for the removal or inactivation of cercariae, by processes including chlorination, filtration, UV disinfection, heating, and water storage. This is the first review of its kind and provides a concise summary of what is known to-date regarding water treatment against cercariae of different *Schistosoma* species. The review also identifies gaps in knowledge and provides crucial and timely guidance for the control and elimination of schistosomiasis, by highlighting the requirements for designing effective and sustainable water infrastructure for schistosomiasis-endemic regions.

## Introduction

Schistosomiasis is a water-borne helminthic disease caused by schistosomes, which are parasitic worms. Infection occurs through dermal-contact with cercaria-infested freshwater. Cercariae are released by snails infected with miracidia which hatch from the eggs in human urine and feces. Despite efforts to control the disease, it remains a major public health problem. Disability-Adjusted Life Year (DALY) estimates have in fact increased over the past 20 years, exceeding 3.3 million DALYs according to the Global Burden of Disease Study 2010 [1, 2]. In 2012, the World Health Assembly (WHA) adopted resolution 65.21 aiming for the elimination of schistosomiasis. This resolution emphasizes the need for multi-sectoral rationale including chemotherapy, strengthening health systems, WASH, and snail control. The need for an integral approach to tackling schistosomiasis has been echoed by many researchers [3–8].

Schistosome cercariae are approximately 500 μm in length, up to 64 μm in width, and are released into freshwater by intermediate host snails [9, 10]. These snails can shed hundreds of cercariae a day–approximately 200 for *S*. *haematobium*, 250–600 for *S*. *mansoni*, and 15–160 for *S*. *japonicum* [11, 12]. Schistosome cercariae are attracted to the human host through skin chemicals, temperature gradient, and turbulence [13, 14]. They are non-feeders, and depend on endogenous glycogen reserves [15]. Cercariae have a forked tail which they use to propel forward and penetrate the host skin. Inside the host, cercariae develop into schistosomula, which eventually develop into schistosomes.

The full schistosome lifecycle was first described in 1908 [16], and interest grew subsequently as troops fighting on African territory were becoming infected [17]. Initially, the control measures focused on mollusciciding and preventing contact with contaminated water (such as described in the War Memoranda from 1919 [18]). The enthusiasm for snail control led to the development of molluscicides (pesticides against snails), which were used to kill all schistosome lifecycle stages in water [19]. The research on water treatment and snail control was slowed by the development of effective orally-administered drugs in the late 1970’s [17, 19]. Ever since, chemotherapy has been the focus of schistosomiasis control programs [8].

The schistosome lifecycle can be cut through chemotherapy, intermediate host control, and WASH. The orally administered drug praziquantel is used to treat schistosomiasis by killing adult worms in the human host. However, it does not prevent reinfection. Snail control reduces the number of intermediate host snails, and is commonly applied through mollusciciding. Although snail control has been shown to reduce disease burden, there are environmental risks associated with molluscicides [19–21]. Universal sanitation aims to prevent eggs found in human feces from entering water bodies, thereby lowering the number of miracidia. However, Grimes *et al*. found no significant association between school sanitation adequacy and *S*. *mansoni* infection intensity in a national survey in Ethiopia [22]. The positive effect of sanitation may only be realized when the entire community adopts the sanitation infrastructure; one miracidium is sufficient to infect a snail, which may release up to 20,000 cercariae in its lifetime, and as a result, only few miracidia are needed to maintain the schistosome lifecycle [12]. Increased hygiene may also impact schistosomiasis control due to the toxicity of soaps to miracidia and cercariae. Use of soaps has been linked with lower prevalence, especially in women who are more exposed to activities such as doing laundry which use soap [23]. Similarly, access to safe water has been found to reduce schistosomiasis prevalence (safe water implying cercaria-free water), as it reduces skin contact with contaminated water [24–26].

The options for providing cercaria-free water are either treating the water, or providing an alternate safe source of water (such as boreholes or rainwater). In endemic regions, there is often no safe alternative water source, and hence water treatment is required to provide a safe water supply. Access to safe water reduces the contact with cercaria-infested water, and may also reduce the risk of water being contaminated with human excreta, as people are spending less time at transmission sites.

Water treatment can provide safe water for domestic use such as washing, laundry, and bathing—activities which are associated with long water-contact periods, and therefore higher risk of schistosomiasis contraction. The community may still have to collect water from transmission sites in order to treat it, but water collection only exposes small areas of the body for short periods and is therefore likely to be less critical. Providing a community with water treatment infrastructure may consequently remove household and recreational activities from transmission sites. This has been demonstrated by numerous studies which observed significantly lower infection rates following the installation of water treatment plants or water recreation areas [24–27]. Water treatment infrastructure can also provide safe water for animals which act as reservoir hosts, particularly for *S*. *japonicum*, and which can play a significant part in transmission [28, 29].

Water treatment infrastructure should not replace other interventions, such as preventive chemotherapy or snail control. On the contrary, it should be implemented alongside chemotherapy-based and behavioral change programs to reduce water contact and thereby minimize the likelihood of reinfection following drug administration. Occupational activities such as fishing and sand harvesting rely on natural water bodies, and exposure to contaminated water will not be affected by the introduction of a safe water supply. Alternative preventive measures may be needed to reduce such forms of exposure (e.g. boots and gloves for fishermen). Even with the provision of safe water, the community may continue to access transmission sites for reasons including preference for untreated water, overcrowding, or lack of privacy at the safe water source, so health education and campaigns to raise awareness of the dangers of exposure to infested water should always accompany water infrastructure implementation. While eliminating contact with infested water bodies completely and forever may be unachievable, strategies could at least be targeted at reducing this contact during and after PC programs, to reduce reinfection rates and hopefully allow a move towards complete elimination of infection in the community.

Water treatment infrastructure also needs careful planning to ensure the technology is sustainable and acceptable to the community. Firstly, however, research is needed to determine which water treatment processes are effective at removing or inactivating cercariae, which is the motivation for this systematic review.

## Methods

The systematic review follows the guidelines of the Preferred Reporting Items for Systematic reviews and Meta-Analyses (S1 Fig). Four databases were searched for the review: Web of Science, PubMed, The British Library, and Google Scholar. All languages and document types were included in the searches, which took place between 7 – 9^th^ April 2017. Given the nature of the topic, many old papers dating from the early 20^th^ century are relevant to this review. However, the oldest paper found in the first three databases dated from 1930. Therefore, Google Scholar was searched from inception to 1950 to ensure that all documentation was included since the discovery of the full disease cycle in 1908. The search dates and results are summarized in Table 1.

**Table 1:** Summary of databases, search limits and paper counts.

Database	Timespan searched	Oldest paper	Number of results
Web of Science	Inception–Present	1970	282
PubMed	Inception–Present	1946	189
British Library	Inception–Present	1930	592
Google Scholar	Inception– 1950	1922	25

The databases were searched for any combination of common schistosomiasis and water treatment terms in the title. A full list of search terms and protocol can be found in S1 Supporting Information.

### Classification criteria

The first and second authors independently classified the papers and extracted relevant information. A classification system that assigns a code to each paper was developed to reduce bias in the classification process (S1 Table). First, duplicates were removed. Then, titles were classified and papers were excluded when titles indicated that they were not about water treatment or cercariae (such as code 4 –this paper is about diagnostic tests). Abstracts of the remaining papers were read, and excluded abstracts were classified (such as code 11 –this paper is primarily about shedding cercariae, not the survival of cercariae). Finally, the remaining papers were read in full and classified. Papers with code 13–19 were used for the systematic review as these papers discuss the effect of water treatment on schistosome cercariae. All codes and papers (included and excluded) are listed in S1 Table, and S2 Fig depicts the flow diagram of the selection process. Full texts were sought from the Imperial College London Library, The Wellcome Library, and the British Library. Papers in other languages were translated by Imperial College London students, and the results discussed with the first author. The two assessors met to compare and discuss the classifications, and any discrepancies were resolved.

### Data extraction

The assessors summarized the papers and extracted specific information regarding each treatment method. To ensure that the same data were extracted, a table with variables relating to each water treatment method was developed (S1 Table). The assessors reviewed each other’s summaries to ensure the correct information was captured.

## Results and discussion

In total, 1088 papers were found by searching the four databases. After removing duplicates, the remaining 520 studies were classified according to the codes in S1 Table. After reading the titles and abstracts, 75 studies were classified to be read in full, however 11 studies could not be acquired. Additional papers were added to the review if they were referenced in the original papers in a way that suggested that they were about human schistosomiasis and water treatment: nine papers were added in this way. Finally, 67 papers were used for the results of this review, and the flowchart in S2 Fig summarizes the classification process.

### Assessing presence, motility, infectivity, and viability of schistosome cercariae in water samples

The studies in this review evaluated the effect of water treatment processes on cercariae, however the measure of effectiveness varied significantly between papers. There were four main aspects that authors assessed–the presence, motility, infectivity, and viability of cercariae. A range of methods exist to evaluate these aspects (summarized in Table 2) with differing results.

**Table 2:** Summary of methods used to assess the effectiveness of water treatment methods.

Cercarial aspect	Technique	Definition
Presence	Microscope	Used in filtration studies to examine if cercariae can pass filter medium.
Motility	Microscope	Evaluates cercarial movement. Time of complete cessation is taken as time of death.
Infectivity	Microscope (examine skin attachment/ penetration) Biosensor	Ability to attach to, and penetrate skin. (Infective cercariae do not necessarily develop into schistosomes.)
Viability	Perfusion of adult worms Fluorescein Diacetate (FDA) method	Ability to penetrate skin and develop into schistosomes, thereby causing schistosomiasis.

Presence of cercariae is used to assess the effectiveness of filtration. Microscopes are used to detect cercariae in water or on a recovery filter. Cercariae may be stained with a dye prior to being filtered to aid with the detection. Some studies fixed cercariae before filtration to facilitate the counting [30, 31]. This may affect results as cercariae are highly mobile and may be able to move through pores that dead cercariae would not pass through [31]. In addition, fixatives have been found to make cercariae sticky, thereby increasing the effectiveness of the filter [31].

For water treatment methods besides physical filtration, the motility of cercariae was most commonly used to assess the effectiveness of water treatment. Cercarial movement is observed under the microscope, often with dyes to facilitate observations. Initially, cercariae in treated water slow their movement, sink, and turn opaque and fluffy. They may lose their tail before the complete cessation of movement [32]. Eventually they may disintegrate, which is a sign of mortality. Most authors used motility as a measure of death. However, it has since been found that immotile or even tailless cercariae may still be infective [33], suggesting that the death of cercariae may have been determined prematurely with the motility measure. Conversely, it is also possible that cercariae that are motile are no longer infective. Cercariae of varying human schistosome species were found to have a functional longevity (time that cercariae are alive but not infective) of 50% of their survival time [34]. This would affect experiments that run longer than the functional longevity.

Infectivity can be used as a measure for testing the cercarial ability to penetrate skin. It is crucial to note that infective cercariae may die before developing into schistosomula or schistosomes, and thereby not necessarily lead to schistosomiasis infection. The attachment of cercariae to skin is visually evaluated under the microscope, or cercariae are counted before and after exposure to the host, the difference assumed to have penetrated the skin. Animal testing on mice, rats and guinea pigs is common, but cultured cells and human skin donors have also been used. Cercariae are able to determine between different types of skin, and hence the results may differ depending on the skin used [35]. Bartlett *et al*. showed that cultured primary keratinocytes are an alternative to animal testing, as the attachment rate is similar to that of human skin [36]. A promising method for assessing cercarial infectivity is a biosensor, as has been developed by Webb *et al*. [37]. Schistosome cercariae secrete elastase when penetrating the host skin, and the biosensor communicates the detection of this elastase through a loss of color.

Only viable cercariae can complete the lifecycle, and therefore transmission is cut if cercariae are rendered non-viable. Cercariae are considered viable if they can infect the human host and develop into schistosomes. Animal testing is often used to determine if cercariae developed into worms. The number of worms and severity of infection can only be determined by perfusing animals for worm counts. Another method for testing the viability of cercariae is the fluorescein diacetate (FDA) method. Viable cercariae are stained green as they take up and retain the chemical, whereas non-viable cercariae lose the green stain due to lacking esterase activity [33]. This method was initially developed to test schistosomula viability [38], and has since been used to test cercarial viability [33].

Ultimately, cercarial infectivity and viability are of greatest interest, as this determines if cercariae can infect a human host with schistosomiasis. Immobile cercariae may still be infective if in direct contact with human skin [33]. Similarly, infective cercariae may not develop into schistosomula or schistosomes and hence are not viable [33]. However, if cercariae are not infective or not viable, schistosomiasis will not be transmitted.

### The effect of water storage and water temperature on schistosome cercariae

Leiper (1915) was the first to study the longevity of schistosome cercariae and found that they could survive up to 36 hours in water [39]. A more conservative estimate was used by the Great Army Medical Services in 1919 who suggested that troops fighting in tropical areas should store water for 48 hours to render it safe [18]. More recent studies found that cercariae live between 10 and 40 hours in natural conditions where water temperature varied between 20 and 30°C [15, 40, 41], but at lower temperatures their lifespan has been shown to exceed 100 hours [32]. It is now commonly accepted that cercariae can survive up to two to three days in environmental conditions [42]. Cercariae are non-feeders and depend on internal, non-renewable glycogen levels. As a result, their glycogen content decreases exponentially post-emergence [15]. However, energy levels do not correlate directly with infectivity: Whitfield *et al*. demonstrated that cercariae remain highly infective for up to nine hours, despite glycogen levels decreasing continually [43]. Differences in cercarial survival may be due to water temperature and water quality.

In 1966, Frick and Hillyer tested the effect of various water media on cercariae, and found that cercariae were immobile in four hours in deionized and steam-distilled water [44]. Mecham *et al*. tested synthetic waters to see which could maintain cercarial life the longest [45]. Soft and distilled water had the fastest cercarial die-off, aquarium water followed, and synthetic hard water could support cercarial life for up to 50 hours (pH, temperature and dissolved oxygen were controlled). Similarly, Asch showed that cercarial lifespan decreases with the addition of sodium chloride, and increases when adding glucose [40]. Overall, these studies demonstrate that the water matrix (the components of the water) can significantly affect results.

In 1911, the impact of temperature on schistosome transmission was first described. Studies found that the disease was acquired from thermal springs in Gafsa that reached temperatures of 45°C, but not from nearby hot springs that were at least 50°C [39, 46, 47]. Since this discovery, it has been shown that the lifespan of cercariae decreases with temperature, when considering the same storage times [15, 48]. This can be due to thermal intolerance or energy depletion. At temperatures exceeding 45°C, extreme temperature causes direct cercarial mortality, known as the thermal death point. The thermal death points for different schistosome species are summarized in Table 3. The point of death was visually examined through cessation of movement. At lower temperatures (below 45°C), increased cercarial death is a consequence of energy depletion. Cercarial lifespan is dependent on glycogen reserves and the rate of utilization, which is affected by temperature [49]. As temperature rises, cercariae become more active, indicated by greater displacement [50] and hence deplete their glycogen levels faster, resulting in shorter lifespans [15].

**Table 3:** Thermal death points for *Schistosoma* species.

Author (year)	Species	Water temperature (°C)	Time until zero surviving cercariae
Krakower (1940) [32]	*S*. *mansoni*	40	6 hours
		42	2 hours
		45	30 minutes
Lawson and Wilson (1980) [15]	*S*. *mansoni*	45	30 minutes
Takaka (1924) [53]	*S*. *japonicum*	45–46	5 minutes
Porter (1938) [52]	*S*. *haematobium*	45	4 hours
	*S*. *haematobium*	50	Instant
Leiper (1915) [39]	-	50	Instant
Khalil (1924) [47]	-	50	Instant
Jones and Brady (1947) [48]	*S*. *japonicum* and *S*. *mansoni*	50	3 minutes
		55	1 minute

**Fig 1:**
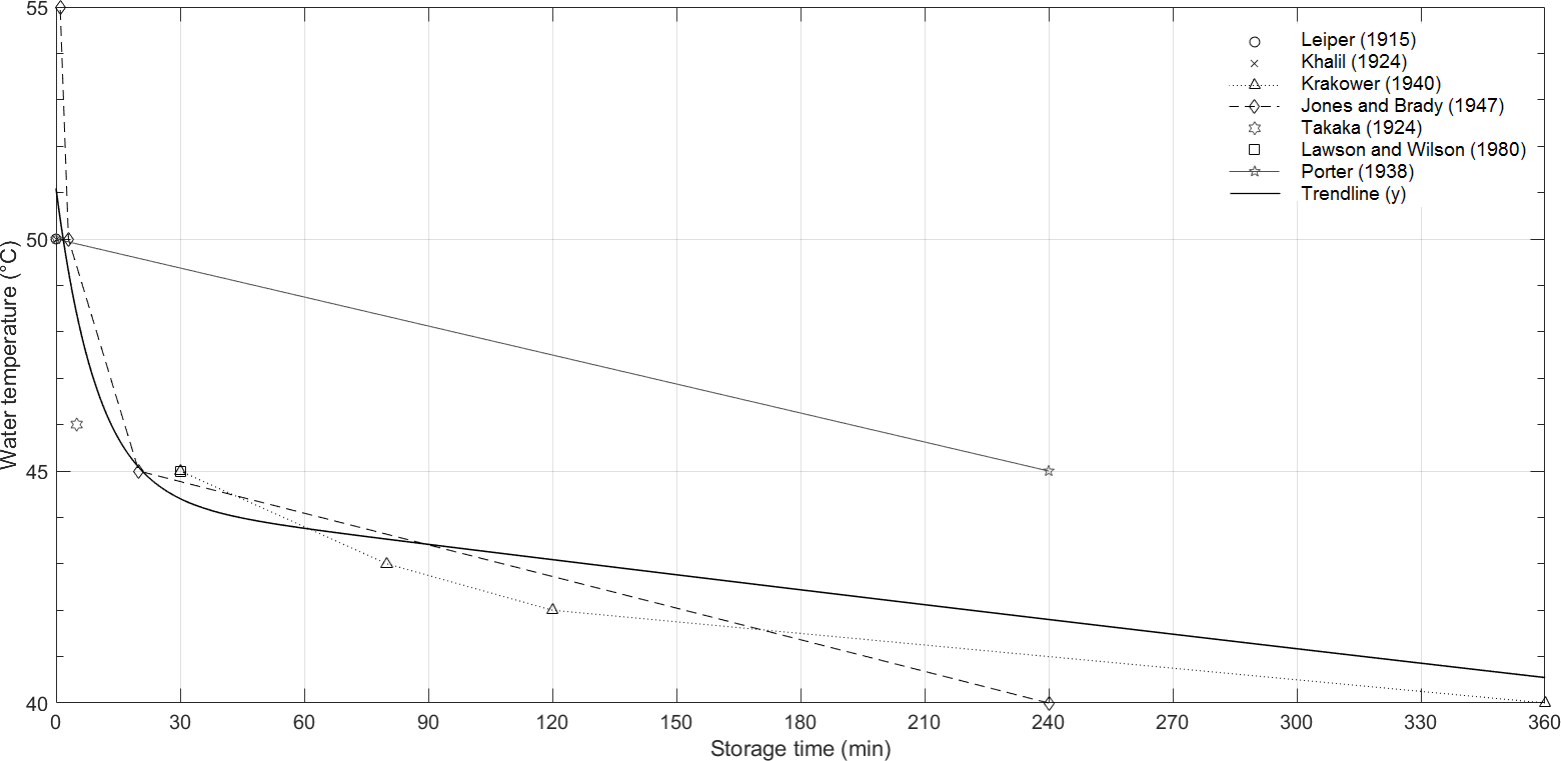
Water temperatures and storage times required to reach zero cercarial survival (measured when all cercariae are immobile). Species are listed in Table 3.

Overall, it can be concluded that the time needed to reach zero cercarial survival decreases exponentially with temperature, as shown in Fig 1. An exponential regression model was fit to the data with an R^2^ of 0.81, indicating a moderately good fit. Cercarial survival is expected to decrease exponentially with temperature, as the depletion of glycogen is also exponential, and the rate of biological processes often increases exponentially with temperature [51]. However, only 14 data points were found and therefore more data are needed to improve the regression model. Cercariae died within minutes at 50°C, and within hours at temperatures below 44°C, though there may be some variation between species. In 1938, Porter carried out the only detailed experiments on *S*. *haematobium* cercariae survival with temperature [52]. The limited results indicate that the species is more resistant at 45°C than *S*. *mansoni*, however more data are needed to verify these results [52]. External factors such as darkness, oxygen or pH (pH 4.6–10) did not appear to affect cercarial longevity [32, 48].

The effect of temperature on cercarial infectivity (ability to penetrate host skin) and viability (ability to develop into schistosomes) follows a similar trend. It has been shown that significantly more cercariae die during host penetration at extreme temperatures (below 10°C and above 40°C) than at 25–27°C for *S*. *mansoni* and *S*. *haematobium* [54]. Similarly, worm recovery increased with temperature to a maximum at 24–28°C for *S*. *mansoni*, followed by a steep decrease [55, 56]. In 1964, Foster was the first to study the relationship between temperature and infectivity, and found that in environmental conditions (18–30°C) temperature did not affect skin penetration and maturation to schistosomula [57]. Similarly, Lee *et al*. found that attachment is more dependent on cercarial age than environmental temperature (20–30°C) [14]. The effect of temperature on the functional longevity of cercariae remains to be studied.

### The effect of water filtration on schistosome cercariae

Filtration is an effective water treatment method that works by physically retaining particles while allowing water to pass. Filters can retain solid matter much smaller than the pores of the filter material due to mechanical and biological processes. Granular filters, which use sand or other granular material, are suitable for use in less developed areas as the filtering media is generally locally available and no external power input is required to pass the water through the filter.

Initial studies on sand filters found that they were unable to remove cercariae from water, and troops were advised to use Doulton candles instead [18, 39, 58]. Nonetheless, Kawata showed that cercariae can be removed from water using sand with an effective size of 0.3 mm or less, and sand depth of 1.2 m [59]. Two studies confirmed that vertical sand filters are effective, however the discrepancy with earlier studies may be due to the heavy dependence on experimental conditions [60, 61]. Primarily, the size of filters varied significantly. Kawata and Fadel tested filter depths up to 1.2 m and 1.4 m respectively with positive results [59, 61], whereas Witenberg and Yofe tested filters up to 0.75 m depth, which could explain the passing of cercariae [58]. Studies found that slow filtration (0.04–0.19 m/h) retained more cercariae than more rapid filtration (0.27–0.4 m/h), as cercariae were not washed through the filter [59, 61]. These studies also showed that conditioning of filters, which develops a biologically active *Schmutzdecke* (biological layer formed on the surface of a sand filter), can improve the overall efficacy of sand filters. The filtration results are summarized in Table 4.

**Table 4:** Summary of experimental conditions in filtration studies. All studies used *S*. *mansoni* cercariae.

Author (year)	Filter medium	Grain size (mm)	Filter depth (m)	Flow rate (m/h)	Cercaria density (l^-1^)	Conditioning	Cercariae recovered in effluent
Leiper (1915) [39]	Sand	Desert sand	0.10	-	-	No	Yes
Leiper (1916) [62]	Sand	Fine sand	0.76	-	-	Yes	Yes
Wagner and Lanoix (1959) [60]	Sand	Fine sand	0.60	0.22	-		No
Kawata (1982) [59]	Sand	0.20–0.30	1.2	0.12–0.4	2000	1–2 hours	No
Fadel (1993) [61]	Sand	0.18–0.37	0.80–1.40	0.19–0.27	10,000	Up to 40 days	No
Witenberg and Yofe (1938) [58]	Sand	-	0.10–0.75	-	-	Yes	Yes
Benarde and Johnson (1971) [63]	Sand	0.19–0.35	0.95	0.36–1.8	290–15,800	No	No
Jones and Brady (1946) [64]	Diatomaceous silica	-	-	2–39	Up to 4200	No	No

In vertical filters, water flows from top to bottom (as opposed to horizontal sand filters in which water moves laterally downwards from one side to the other). Benarde and Johnson tested horizontal sand filters, which are often constructed in channels filled with sand [63]. Cercariae were unable to pass horizontal sand filters with 0.35 mm effective grain size and 0.9 m bed depth. These filters seem to achieve better results than vertical sand filters, the hypothesis being that the natural movement of cercariae is vertically up and down, so using a filter where water flows horizontally helps filter out cercariae [63]. Further research is needed to confirm this hypothesis.

Generally, filters used one grain size, but a filter with layers of different sized sand may prove to be beneficial and reduce the overall depth of the filter. Pre-filters would also reduce clogging and allow the filters to be used in the field with turbid water. Further research is needed to test the effect of sand size, flow rate, filter depth, filtration volume and water quality on sand filtration as a method to completely remove cercariae from water.

Other filter materials that have been tested include diatomaceous silica, a naturally occurring rock that is often used in filtration as a powder. Jones and Brady tested three types of diatomaceous silica filters and recovered no cercariae from the effluent, although no filter material sizes were specified [64, 65]. Diatomaceous silica has small, irregular pores which helps prevent cercariae from passing through the filter.

Membrane filters have been used for cercariometry (to determine the density of cercariae in water. Micro-fabrics with pore sizes of 23–30 μm have successfully filtered out cercariae [30, 31, 66–71]. When filtering natural water, several pre-filters were used to prevent the clogging of the filter. While a slow filtration rate was shown to be beneficial in sand filtration, others found that more rapid flow rates immobilized cercariae and prevented them from moving themselves through the membrane [31]. Although experiments varied in cercaria numbers, water volume, water quality, filtration rate and time, the results are consistent.

### The effect of chlorination on schistosome cercariae

Water chlorination is an effective water treatment method that kills most bacteria and viruses. Chlorine is an oxidizing agent and kills pathogens through oxidation. Chlorine disintegrates the cell wall of pathogens, rendering them unviable. In chlorination, a ‘CT’ value indicates the residual chlorine dose (C) and contact time (T) required to inactivate a pathogen, and is calculated as the product of the two variables. CT values have been determined for many waterborne pathogens (see Table 5), and can be used as a process monitoring parameter, i.e. simply ensuring that the required CT is being achieved means that constant pathogen monitoring is not required. Higher CT values indicate a higher chlorine tolerance.

**Table 5:** Chlorine CT values for selected pathogens, adapted from Centers for Disease Control and Prevention (2012) [72].

Pathogen	Residual concentration of chlorine (mg/l)	Chlorine contact time (min)	CT (mg-min/l)	% Inactivation	Temperature (°C)	pH
*Escherichia coli*	0.5	<0.5	<0.25	99.99%	23	7
*Salmonella typhi*	0.05	20	1	99.2%	20–25	7
*Vibrio cholerae (rugose strain)*	2.0	20	40	99.99%	20	7
*Cryptosporidium parvum*			15,300	99.9%	25	7.5
Hepatitis A	0.41	<1	<0.41	99.99%	25	8

The effect of chlorine on schistosome cercariae has been researched throughout the past century, with varying conclusions. In 1920, Mahnson-Bahr and Fairley found that some cercariae (unknown percentage) were still alive after 2.5 hours in water containing 4 mg/l of chlorine [10], whereas Magath found that 0.2 mg/l after 30 minutes was sufficient to kill all cercariae [73]. More recently, the WHO stated that a chlorine residual of 1 mg/l after 30 minutes is sufficient to render cercaria-free water [74]. Table 6 summarizes some of the chlorination results found in this review. Note that the contact time is the time until inactivation of all cercariae (100% kill), which in all studies was measured by complete cessation of movement of all cercariae in the sample.

**Table 6:** Chlorine concentrations and contact times required to kill all schistosome cercariae in water (measured by cessation of movement).

Author (year)	Species	Chlorine form	Chlorine dose (mg/l)	Residual chlorine dose (mg/l)	Contact time (min)	Calculated CT value (mg-min/L)	Water characteristics
Leiper (1915) [39]	-	-	11	-	Instantly	-	-
	-	-	6	-	3	-	-
Leiper (1916) [62]	-	-	2	-	30	-	
Manson-Bahr and Fairley (1920) [10]	-	-	No cercaricidal effect noticed at 4 mg/l and 2.5 hours			-	-
Blackmore (1928) [75]	*S*. *mansoni*	Chloramine	1	-	5	-	Tap water
	*S*. *mansoni*	Bleach	1	-	15	-	Tap water
	*S*. *mansoni*	Bleach	1	-	5	-	Unclarified Nile water
	*S*. *mansoni*	-	-	1	30	30	-
Griffiths-Jones *et al*. (1930) [76]	*S*. *mansoni*, *S*. *haematobium*	-	1	-	180	-	Filtered water
Witenberg and Yofe (1938) [58]	*S*. *mansoni*, *S*. *haematobium*	-	1	-	Instantly	-	Clarified river water
	*S*. *haematobium*	-	0.8	-	27	-	Clarified river water
Braune (1942) [77]	*S*. *mansoni*	-	1.5	-	15	-	Tap water
Magath (1942) [73]	*S*. *mansoni*	Calcium hypochlorite	-	0.1	30	3	Rain water
	*S*. *mansoni*	Calcium hypochlorite	1	-	6	-	Rain water
González *et al*. (1945) [78]	*S*. *mansoni*	Calcium hypochlorite	1	-	7	-	Redistilled water
	*S*. *mansoni*	Calcium hypochlorite	0.5	-	18	-	Redistilled water
	*S*. *mansoni*	Calcium hypochlorite	-	0.5	20	10	-
Jones and Brady (1947) [65]	*S*. *mansoni*	Chloramine	1	-	10	-	Raw surface water
	*S*. *mansoni*	Calcium hypochlorite	-	1.67	10	16.7	Buffered distilled water
	*S*. *japonicum*	Calcium hypochlorite	1.3	0.5	10	5	Buffered distilled water
	*S*. *japonicum*	Calcium hypochlorite	1.4	0.75	10	7.5	Aquarium water
Wagner and Lanoix (1959) [60]	*-*	Chloramine	-	1 mg/l at 1 min	10	-	Filtered water
	*-*	Calcium hypochlorite	-	0.75 mg/l at 30 min	20	-	Unfiltered water
Frick and Hillyer (1966) [44]	*S*. *mansoni*	Sodium hypochlorite	0.3	-	30	-	Tap water pH 5
	*S*. *mansoni*	Sodium hypochlorite	0.6	-	30	-	Tap water pH 7.5
	*S*. *mansoni*	Sodium hypochlorite	5	-	30	-	Tap water pH 10
World Health Organization (2000) [74]	*-*	-	-	1	30	30	-

**Fig 2:**
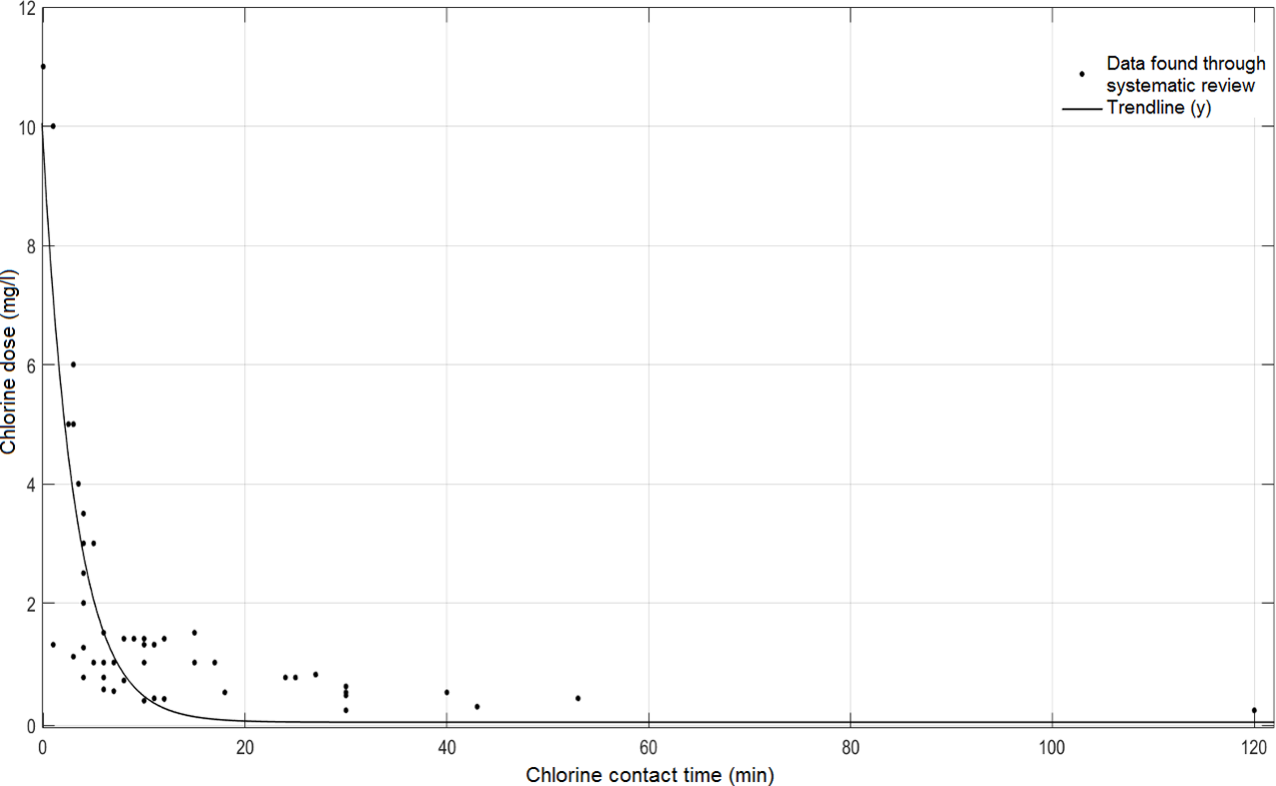
Lethal chlorine doses for killing 100% of schistosome cercariae. All studies used motility as a measure of death, and recorded the time at which all cercariae were motionless. Chlorine doses are plotted here because most studies did not report the residual chlorine concentrations. Species are listed in Table 6 and shown in S3 Fig.

The data sets of all studies have been plotted in Fig 2 to show the effect of varying chlorine doses on cercariae (a more detailed graph is shown in S3 Fig). Note that these are doses of chlorine (the chlorine added to the water), not residual chlorine concentrations in the water (accounting for the reaction of chlorine with other water constituents); the lack of reporting of the chlorine residual concentration is a major weakness of many prior studies.

It is evident that in all studies the effect of chlorine increased exponentially with chlorine dose. High doses, generally above 1.5 mg/l, killed all cercariae within minutes. The data follows a clear trend and can be fit to an exponential regression model with R^2^ of 0.68 (Fig 2). Data is consistent at low lethal chlorine doses, but there are discrepancies at short inactivation times. This is particularly the case for inactivation times less than 30 minutes (see S3 Fig) where the lethal chlorine dose at 3 minutes, for example, varies between 1.1 mg/l and 5 mg/l. This is enforced by the variation of calculated CT values in Table 6, which vary from 3 to 30 mg-min/l. The variation may be due to differing experimental conditions, such as pH, temperature, water quality (e.g. organic matter which will react with chlorine), the form of chlorine used, chlorine measurement techniques, and schistosome species, or because of incomplete mixing of the chlorine into the water at very short contact times.

The type of water and water quality used in the experiments varied greatly. This is noteworthy, as water temperature and pH significantly affect the CT value. When sodium hypochlorite is added to water, it reacts to form hypochlorous acid. It may react further, especially under alkaline conditions, to form hypochlorite ions. The latter is a much weaker disinfectant and as a result, more chlorine is needed at higher pH to achieve the desired level of disinfection. Generally, a higher CT value is needed at lower temperature and higher pH. Lower temperature results in lower rates of chemical disinfection. In 1942, Braune tested ways to increase the efficiency of chlorine against cercariae [77]. He found that the addition of citric acid heavily accelerated the chlorine disinfection process, and suggested adding 100 mg/l of citric acid to the water. Frick and Hillyer followed in 1966 with a detailed study on the effect of chlorination on cercariae with respect to water temperature and pH [44]. They found that pH had a much greater effect on chlorine efficiency than temperature; in tap water at 20°C, *S*. *mansoni* cercariae were inactivated in 30 minutes with chlorine concentrations of 0.3 mg/l at pH 5, 0.6 mg/l at pH 7.5, and 5 mg/l at pH 10.

The chlorine demand is the difference between the amount of chlorine added to water and the residual chlorine after a given contact time. Generally, turbid water has a higher chlorine demand and consequently the CT value may be expected to increase with turbidity. Pathogens may attach to particulate matter in the water, which may encapsulate them. For this reason, CT values are usually calculated for low turbidity water, and values are then doubled in the field to account for the variability in water quality [72]. Experimental studies should account for the chlorine demand of the waters that they are testing and not simply report the chlorine dose added to the water. It is critical to measure the residual chlorine levels as chlorine decomposes rapidly, and when added to water reacts with chemicals, organic matter, and pathogens. These reactions remove some of the chlorine, hence the residual chlorine is lower than the chlorine dose added. Water that is highly contaminated therefore requires more chlorine to inactivate pathogens, to overcome the chlorine demand of the water matrix. Some authors did reference the available chlorine residual, but the time of measurement did not always coincide with the inactivation time [76, 77].

The form of chlorine used in experiments included chloramine, chlorine gas, bleaching powder, and calcium hypochlorite. These may have differing cercaricidal effects, even when used in equal available chlorine doses. Authors have reported that chloramine was less effective than chlorine, but more powerful than gaseous chlorine, and that the method of preparing chlorine also affected the results [58, 76]. Similarly, the stability of chloramine varied depending on how it was prepared. The Great Britain Army Medical Services advised against using water chlorination as a means to disinfect cercaria-infected water because chlorinated lime degraded too quickly, and the presence of sufficient free chlorine could not be ensured [18]. In addition, the method for measuring residual chlorine may be a source of error, as the DPD titration, orthotolidine method, and colorimetric test kits used in the studies have varying accuracies [79].

The species appear to impact results. *S*. *haematobium* has been found to be more sensitive to chloramine, and *S*. *mansoni* more sensitive to gaseous chlorine [58, 76]. Out of the 12 chlorine studies identified in this systematic review, only three experimented with *S*. *haematobium* or *S*. *japonicum* cercariae, stressing the need to research these *Schistosoma* species. In addition, it was only these three studies that simultaneously tested two species [58, 65, 76]. They found that *S*. *haematobium* and *S*. *mansoni* had differing sensitivities to chlorine, which highlights the need to conduct further research on all species under a variety of water matrix conditions.

The age of the cercariae may also play an important role in chlorination, but no research has evaluated this. Generally, studies used freshly hatched (<1 hour) cercariae. Sproule stated that fresh cercariae were more resistant to chlorine than cercariae older than 1 hour [80]. This could be due to cercariae hatching within mucus from the snail [43], which may act as a protective layer.

All authors used motility as a measure of death, and the points on Fig 2 show the time until the last cercaria stopped moving. As previously mentioned, cessation of movement does not necessarily indicate death, so the values in Fig 2 may be an underestimation of lethal chlorine doses.

In water disinfection studies, log-inactivation is commonly used to indicate what percentage of pathogens has been inactivated. As the lethal chlorine doses were recorded in previous studies when all cercariae were dead, the experiments reported a 100% kill. However, due to the small cercaria samples used (i.e. 100 or fewer cercariae), the highest log-kill that could be claimed was a 2-log kill (99% kill). Samples exceeding 1000 cercariae would need to be tested to claim a 3-log inactivation (99.9% kill), which may be challenging if evaluating the motility. Also, survival curves for cercariae follow a reverse-sigmoid form [15], which suggests that if a small sample is used, the results may be less reliable. This could explain the differences between Witenberg and Yofe, and Frick and Hillyer, as the former tested up to 200 cercariae [44], whereas the latter used as little 20 cercariae in experiments [58].

### The effect of ultraviolet disinfection on schistosome cercariae

UV disinfection uses radiation in the UV wavelength range to inactivate microorganisms. This range is often referred to as germicidal, and spans wavelengths of 200–300 nm. UV is absorbed by the DNA and RNA of microorganisms, damaging cell membranes and thereby inhibiting reproduction. The sensitivity of microorganisms to UV is determined by the UV fluence (mJ/cm^2^)–the product of the fluence rate and exposure time [81].

In this review, two types of UV studies were found: immunization studies and water treatment studies. Immunization studies research the possibility of using UV-attenuated cercariae to develop a vaccine against schistosomiasis. The process is as follows: cercariae are irradiated with UV to a level that damages them but that does not prevent host penetration. Irradiated cercariae infect the host by penetrating the skin, but due to the UV damage are unable to develop into adult worms. Nonetheless, antibody production is triggered, and as a result, the host builds up immunity. Lighter, succeeding infections is proof that partial resistance is induced [82, 83]. The other type of UV studies found in this review was water treatment studies. These aim at using UV to disinfect cercaria-infested water and kill or inactivate cercariae. Both types of studies therefore use UV to damage cercariae, their protocol is however very different. Immunization studies irradiate cercariae so that their movement is not impaired, and they are still able to penetrate the host, whereas water treatment studies generally use higher UV fluences to instantly kill cercariae.

The effect of UV on cercariae and worm burden is summarized in Table 7. Cercariae were irradiated with fluences between 3 mJ/cm^2^ and 1890 mJ/cm^2^, all at 254nm, but the direct effect (motility, infectivity) on cercariae was often not evaluated as the focus was on worm burden (viability). Krakower was the first to research the effect of UV radiation on schistosome cercariae and found that cercariae appeared physically injured after 30 minutes, and motionless after 60 minutes exposure to sunlight when kept in a shallow container [32]. UV fluences as low as 3 mJ/cm^2^ have been shown to damage cercariae, slow their movement and reduce their survival [84, 85]. Higher fluences were tested in a series of experiments aimed to identify the UV fluence that triggers the highest level of host immunity [84]. The authors tested fluences up to 60 mJ/cm^2^ and found that 54 mJ/cm^2^ instantly reduced cercarial motility and damaged their physiology by inhibiting glycoprotein synthesis. A further increase to 60 mJ/cm^2^ made cercariae immotile and not infective. It was determined that the appropriate UV fluence for immunization studies was 18–24 mJ/cm^2^, as it does not inhibit cercarial penetration but results in significantly lower worm burden, as confirmed by numerous other studies [86–88]. In addition, the migration potential of adult worms is reduced, hampering their ability to migrate from skin to organs [84, 89]. Overall, cercarial movement, infectivity, and viability decreased with UV fluence.

**Table 7:** Effect of UV disinfection on cercariae and worms. *All fluences at 254nm.

Author	Species	UV fluence* (mJ/cm^2^)	Time (min)	Effect on cercariae	Effect on worm burden
Krakower (1940) [32]	*S*. *mansoni*	Sunlight	30	Severely injured	Not assessed.
	*S*. *mansoni*	Sunlight	60	Killed	Not assessed.
Ariyo and Oyerinde (1990) [85]	*S*. *mansoni*	-	0.05	After 4h, moving cercariae reduced from 69% (control) to 17%	Not assessed.
	*S*. *mansoni*	-	0.17	After 4h, moving cercariae reduced from 69% (control) to 9%	After 4 weeks, worm burden reduced to 11% of control After 8 weeks no worms recovered.
Standen and Fuller (1959) [90]	*S*. *mansoni*	-	4	Killed	Not assessed
Ghandour and Webbe (1975) [82]	*S*. *mansoni*, *S*. *haematobium*	-	<0.3	No effect on movement Mortality during skin penetration more than doubled compared to control	No worms recovered
	*S*. *mansoni*, *S*. *haematobium*	-	0.5	Sluggish appearance, severely damaged	-
	*S*. *mansoni*, *S*. *haematobium*	-	3	Killed (no movement)	-
Ruppel (1990) [84]	*S*. *mansoni*, *S*. *japonicum*	3		Not assessed	50% survival from cercariato worm
	*S*. *mansoni*, *S*. *japonicum*	12		Not assessed	1% survival from cercaria to worm
	*S*. *mansoni*, *S*. *japonicum*	60		Killed	-
Kumagai *et al*. (1992) [86]	*S*. *mansoni*	5		Not assessed	Disturbs migration potential
	*S*. *mansoni*	10		Not assessed	Hampers migration from skin to lungs
	*S*. *mansoni*	18		Not assessed	No worms recovered in lung and liver
Kamiya *et al*. (1993) [89]	*S*. *mansoni*	18	0.67	Not assessed	Migration reduced to skin
Dean *et al*. (1983) [87]	*S*. *mansoni*	19.8	3	Not assessed	No worms recovered
Lin *et al*. (2011) [88]	*S*. *japonicum*	24	1	Not assessed	59% reduction in worm burden
Shi *et al*. (1990, 1993) [28, 29]	*S*. *japonicum*	24	1	Viability reduced to 0.1%	Reduced by 89%. Survival rate of cercaria to worm is 0.01%
Tian *et al*. (2010) [91]	*S*. *japonicum*	24	1	Not assessed	Worm burden reduced by 63.84%
Nakamura *et al*. (1990) [92]	*S*. *japonicum*	300		Not assessed	No worms recovered
Moloney *et al*. (1985, 1987) [93, 94]	*S*. *japonicum*, *S*. *mansoni*	1890	0.75	Not assessed	No worms recovered Host 70% resistant for 40 weeks

The study by Ghandour and Webbe clearly demonstrates the importance of evaluating the different aspects of cercariae (motility, infectivity, viability) [82]. Irradiation for less than 0.3 minutes showed no effect on cercarial movement. However, the impact was visible when cercariae were exposed to skin; increased mortality rates during skin penetration resulted in reduced cercarial infectivity. Furthermore, the authors studied cercarial viability by examining schistosomula migration and worm burden. Most schistosomula died within days and did not develop into schistosomes, and were thus not viable [82, 95]. Similarly, Ariyo and Oyerinde showed that the damaging effect of radiation may not be immediately apparent at low UV fluences [85]. Within the first hour post-radiation, there was no effect on cercarial movement, and the attachment rate ranged between 93% and 100%, similar to the control. Nonetheless, the effect of UV became apparent in the stages following host penetration. Significantly fewer cercariae developed from schistosomula into schistosomes, and those that did had reduced fecundity, indicated by the decrease in egg load and egg viability.

The increased cercarial death associated with UV irradiation may be partially due to the increased water temperature. At the time of the studies, UV lamps emitted significant amounts of heat, easily able to increase the temperature of the water within the running time of the experiment. The risk of temperature affecting results is especially high for studies that used small water samples and long irradiation times. This could explain the positive results by Dean *et al*. who recovered no worms at fluence 19.8 mJ/cm^2^ and 3 minutes [87], whereas other studies using higher UV fluences but shorter exposure times simply achieved a worm reduction [28, 88]. The heat production and changes in temperature of the water were not recorded in the studies.

It appears that *S*. *mansoni* and *S*. *japonicum* are equally sensitive to UV, which may be explained by similar distribution of cercariae in water columns; their cercariae accumulate at the water surface, whereas *S*. *haematobium* cercariae prefer to remain beneath the surface [49]. This characteristic could lead to *S*. *haematobium* cercariae being less damaged by UV radiation, as accumulating at the bottom of the water column provides a protective water layer. Only one study tested *S*. *haematobium*, and three studies simultaneously tested two species, making it difficult to compare the results of all species.

UV radiation has been shown to inhibit the migration within the host, hampering the worms’ ability to migrate from skin to organs [82, 86]. This may be due to the physical damage of worms that develop from irradiated cercariae. One study examined the physical effect of UV on *S*. *mansoni* worms, and found that worms developed from cercariae that were irradiated for 1 minute with a Mineralight lamp (unknown fluence) had lesions, torn tubercles, and lost their spikes. This resulted in sexual anomalies and sterility [96].

The experiments generally irradiated cercariae in a shallow container with tap or distilled water. Therefore, the UV fluence measured at the surface of the water was assumed to be the UV fluence reaching the cercaria surface. This, however, is not the case since suspended particles and water itself absorb UV light, reducing the depth of UV penetration. The turbidity and depth of a water sample increase the UV fluence needed to kill cercariae, and this needs to be considered in future research.

The studies demonstrate that UV radiation damages cercariae, however the lack of measurements and controlled variables makes it difficult to draw concrete conclusions. Numerous studies did not measure the UV fluence or irradiation time, as seen in Table 7. Generally, the fluence was measured at the surface of the water with a UV meter. However, occasionally the distance at which the fluence was measured did not correspond to the surface of the water, and was hence not applicable [90]. The time of taking UV measurements may also affect the accuracy of UV fluences. Some authors took UV readings instantly after turning on the lamp, whereas others allowed the bulb to warm up for 15–30 minutes before taking measurements. Cercariae were irradiated in a variety of containers which included quartz cuvettes, glass slides and well plates, which may also affect the level of UV radiation.

The density of cercariae varied greatly between experiments, and may have affected results as cercariae may shield each other in high densities. It is crucial to carry out experiments under well-defined and controlled UV fluences to truly determine the UV-sensitivity of schistosome cercariae.

### Conclusion

The results of this systematic review indicate that there are many differences in assessing the effects of water treatment processes on schistosome cercariae (i.e. motility, infectivity, viability), making it difficult to directly compare the results of studies that have used different criteria of death. It is necessary to determine at what level of motility cercariae stop being infective and viable. Methods like the biosensor and FDA described in this review may prove to be useful, as they assess the viability of cercarial populations in a water sample, not of each individual cercaria. All treatment methods should be evaluated with the same criterion that ensures cercariae are not infective or viable.

The level of variability in results differs between treatment processes. Previous studies on the effect of water storage and temperature are relatively in agreement, and indicate that water quality and pH do not greatly affect these two treatment methods. Cercariae are killed when storing water for more than one to three days, depending on temperature. The results of other water treatment processes, filtration, chlorination, and UV, are much more variable because the processes are heavily affected by experimental conditions, and require further research to obtain reproducible information. It is evident that the water matrix used in experiments hugely impacts cercaria survival. Most studies conducted experiments using lab-hatched cercariae and tap or filtered water. This builds a foundation for understanding the effect of water treatment processes on cercariae. However, it is essential to also conduct experiments under real water matrix conditions. In addition, water treatment processes should be tested individually as well as in succession of each other, such as filtration followed by chlorination, as this may achieve higher levels of cercaria removal than the treatments applied in isolation.

The variability in findings is likely also due to differences in experimental protocols and discrepancies in measuring key parameters. Chlorination studies often measured different chlorine values, such as chlorine dose, free chlorine at one-minute, or free chlorine at point of cercarial death. As there is no conversion between these measurements, the studies are incomparable. UV fluence measurements often did not correspond to the fluence reaching cercariae in the water. Filtration studies varied immensely in terms of scale and running time, making it difficult to compare the results. Experiments for all treatment processes should measure the variables needed to reliably design water treatment infrastructure.

The review has highlighted the lack of water treatment studies testing *S*. *haematobium* and *S*. *japonicum*. The majority of studies (57%) tested *S*. *mansoni*, 15% *S*. *japonicum*, and 3% tested *S*. *haematobium*. Only 12% of the studies tested more than one species (either *S*. *mansoni* and *S*. *haematobium*, or *S*. *mansoni* and *S*. *japonicum*), making it difficult to draw conclusions regarding their relative resistance to water treatment processes. The remaining 13% did not indicate which species were being tested, primarily older studies.

This review focused on the three main human *Schistosoma* species and no studies about water treatment processes and animal reservoir hosts were found in this review. The only studies involving animals were immunization trials using UV-attenuated cercariae. Animal reservoirs, including domestic livestock, can play a critical role in schistosomiasis transmission, especially of *S*. *japonicum* [28, 29]. Using treated water for livestock water use may contribute to lower infection rates in both humans and animals, and is therefore of medical and veterinary importance.

Implementing water treatment infrastructure could remove household water activities such as laundry, washing and recreational swimming from transmission sites. Depending on the level of treatment required to remove cercariae, other water-borne pathogens may also be inactivated, rendering water safe for drinking (e.g. if chlorination at a sufficient contact time is used). Successfully implementing and sustaining the infrastructure will require careful consideration of issues of equity of access and affordability, and it is crucial that educational programs run alongside the implementation to ensure understanding of the operation and maintenance needs. All five reviewed water treatment processes can be implemented as low-cost household or community-scale water treatment systems, yet the cost is dependent on local conditions such as availability of materials or supply chains. It is important to note that the provision of water treatment infrastructure will not remove occupational water exposure, such as fishing, from transmission sites. Nonetheless, an overall reduction in the population’s exposure to contaminated water may result in lower odds of infection and reinfection following chemotherapy, a relationship that needs to be more carefully studied and quantified. Safe water infrastructure may accelerate progress towards control and elimination of schistosomiasis by being incorporated into PC-based strategies, where it would serve to reduce the need for contact with infested water. We propose the following research priorities:

1. Determine a sensitive and specific measure for cercarial viability or infectivity.
2. Determine the effectiveness of water treatment processes against schistosome cercariae under lab and field conditions, as well as in combination.
3. Develop water treatment guidelines based on the results from water treatment experiments.
4. Examine the non-technical aspects of implementing water treatment infrastructure in a schistosomiasis-endemic region, such as cost, operation, maintenance, ownership and education.
5. Design and develop sustainable water treatment infrastructure based on the guidelines (3) and the social and economic factors (4). This may be on household scale (e.g. household sand filter) or community level (e.g. community storage tank).
6. Develop educational programs to run alongside the implementation of water treatment.
7. Incorporate water treatment infrastructure implementation and water contact awareness campaigns into schistosomiasis elimination programs.
8. Quantify the impact of water treatment infrastructure on human water contact and intensity of infection and reinfection following chemotherapy.

The literature demonstrates that all the reviewed water treatment methods have the potential to effectively remove or kill cercariae and thereby produce safe water. However, the database of required water treatment is insufficient to be able to devise guidelines for the design of water infrastructure for schistosomiasis-endemic regions. As countries target the control and elimination of schistosomiasis, it will be crucial to develop such water treatment guidelines, and to better link activities of the WASH sector to PC-based control programs.

## Supporting information

S1 Fig PRISMA checklist. (DOC)

S2 Fig Flow diagram. Flow chart outlining the selection process for studies in this review.
(PDF)

S3 Fig Lethal chlorination doses and contact times. Data found in the systematic review, showing chlorine doses (up to 6 mg/l) and respective contact time (up to 30 minutes) required to kill cercariae. All studies used motility as a measure of death, and recorded the time when 100% of cercariae were immobile.
(TIF)

S1 Supporting Information Search protocol. Search terms and examples of search strategy for two databases.
(DOCX)

S1 Table Classified included and excluded studies. Classification codes, and a list of included and excluded studies.
(XLSX)
