# Supplementary figures and images for: The effectiveness of water treatment processes against schistosome cercariae: A systematic review
Source: PLoS Negl Trop Dis. 2018 Apr 2;12(4):e0006364. doi: 10.1371/journal.pntd.0006364 (PMC5903662; doi:10.1371/journal.pntd.0006364)

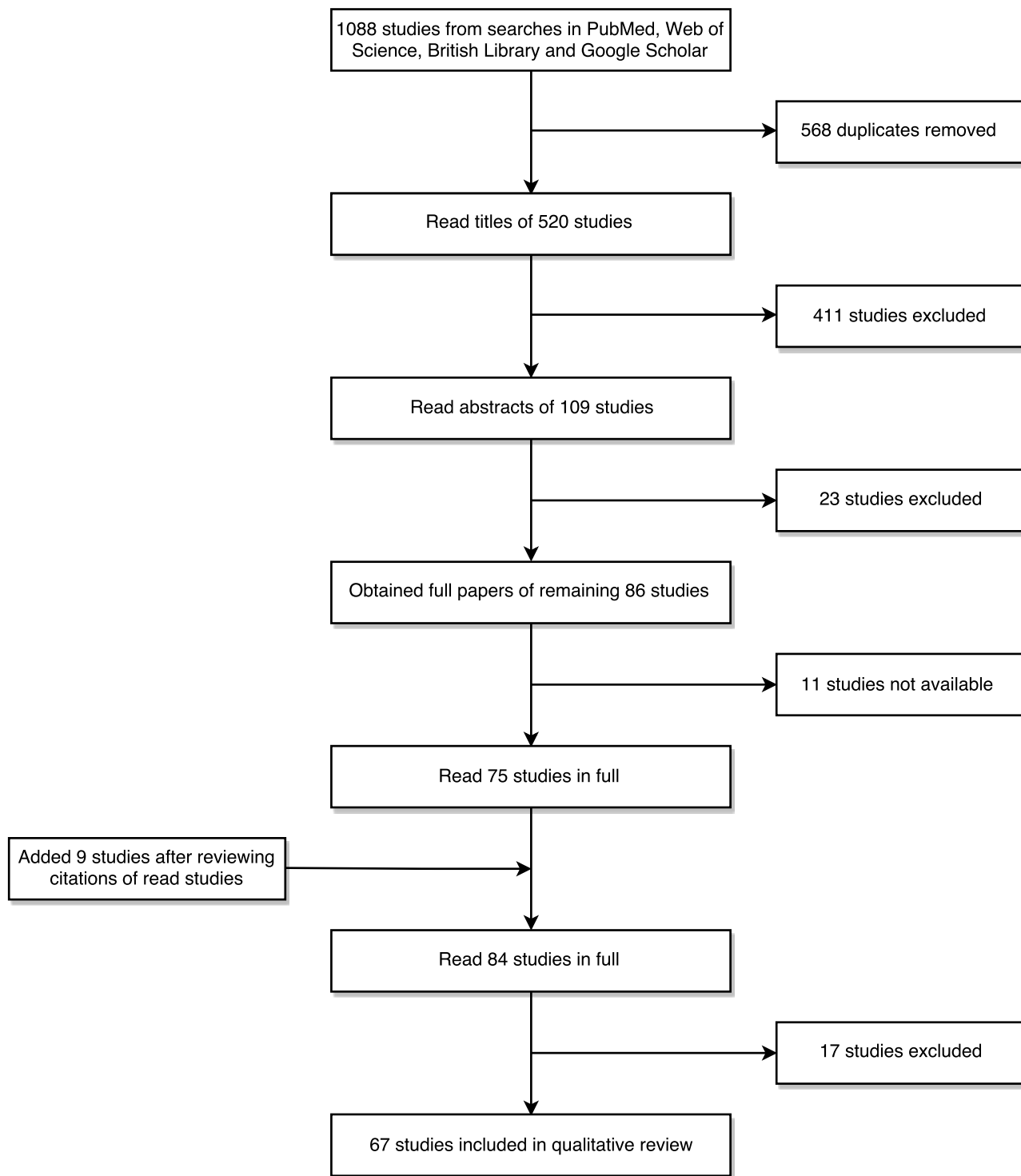

*S1 Fig: Flow diagram outlining the selection process of studies for this review*

Supplement: S2 Fig — Flow chart outlining the selection process for studies in this review. (PDF) [file pntd.0006364.s002.pdf]

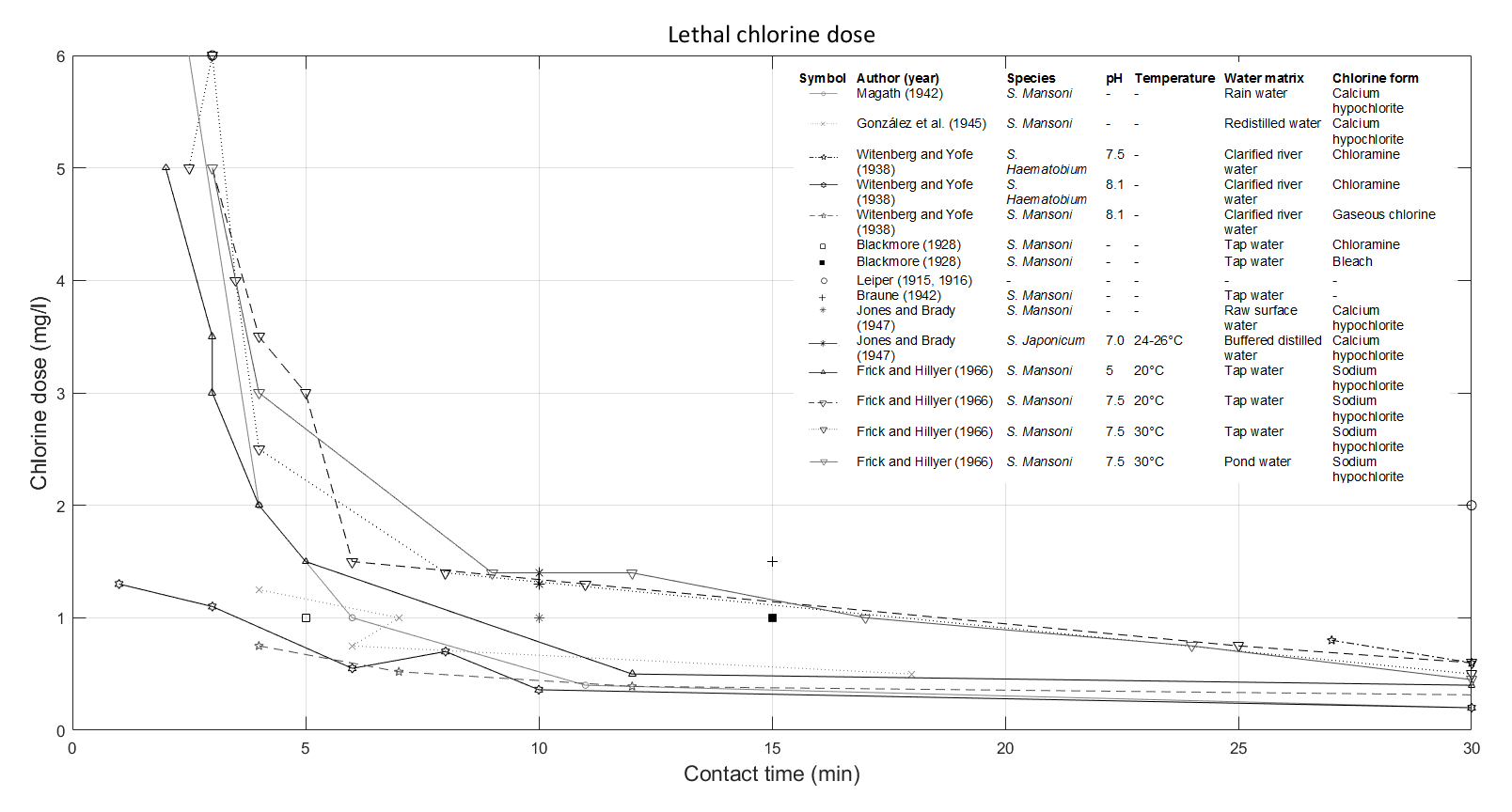

Supplement: S3 Fig — Data found in the systematic review, showing chlorine doses (up to 6 mg/l) and respective contact time (up to 30 minutes) required to kill cercariae. All studies used motility as a measure of death, and recorded the time when 100% of cercariae were immobile. (TIF) [file pntd.0006364.s003.tif]
