# Supplementary material for: The effectiveness of water treatment processes against schistosome cercariae: A systematic review
Source: PLoS Negl Trop Dis. 2018 Apr 2;12(4):e0006364. doi: 10.1371/journal.pntd.0006364 (PMC5903662; doi:10.1371/journal.pntd.0006364)
Supplement: S1 Supporting Information — Search terms and examples of search strategy for two databases. (DOCX) [file pntd.0006364.s004.docx]

**S1 Supporting Information**

**Search terms:**

The schistosomiasis terms were *Bilharz*, Cercaria*, Haematobium, Japonicum, Mansoni, Schistosom*, Snail fever*.

The water treatment terms were *Disinfect*, Water treatment, Purification, Calcium hypochlorite, Chlorin*, Chloramine, Filtration, Filter, Flocculation, Iodine, Sunlight, SODIS, Solar disinfection, Temperature, Storage, UV, Ultra-violet, Ultraviolet*. The asterisk was used as a wildcard to find variations of the exact phrase (i.e. Bilharz* returned searches including Bilharzia, Bilharziasis etc.). The search term Rhizobium was excluded, as it produced many unrelated results.

**Example search strategy:**

For reference, two searches are presented below. The first is for the database Web of Science, the second for PubMed.

TI=(Bilharz* OR Cercaria* OR Haematobium OR Japonicum OR Mansoni OR Schistosom* OR Snail fever) AND TI=(Water treatment OR Purification OR Calcium hypochlorite OR Chloramine OR Chlorin* OR Filtration OR Filter OR Flocculation OR Iodine OR Potassium permanganate OR Sunlight OR SODIS OR Solar disinfection OR Temperature OR Storage OR UV OR Ultra-violet OR Ultraviolet) NOT TS=Rhizobium

Search ((Bilharz*[Title] OR Cercaria*[Title] OR Haematobium[Title] OR Japonicum[Title] OR Mansoni[Title] OR Schistosom*[Title] OR Snail fever[Title])) AND (Water treatment[Title] OR Water purification[Title] OR Calcium hypochlorite[Title] OR Chlorin*[Title] OR Filtration[Title] OR Filter[Title] OR Flocculation[Title] OR Iodine[Title] OR Potassium permanganate[Title] OR Sunlight[Title] OR SODIS[Title] OR Solar disinfection[Title] OR Temperature[Title] OR Storage[Title] OR UV[Title] OR Ultra-violet[Title] OR Ultraviolet[Title]) NOT Rhizobium[Title]
